# Spatial topology and competitive access differentially shape early T cell priming in the lymph node: an agent-based modeling approach

**DOI:** 10.3389/fimmu.2026.1843390

**Published:** 2026-06-02

**Authors:** Laia Vancells, Leopold Green, Nan Kong

**Affiliations:** Weldon School of Biomedical Engineering, Purdue University, West Lafayette, IN, United States

**Keywords:** agent-based modelling, computational immunology, lymph node, T cell priming, dendritic cells, fibroblastic reticular cells, spatial topology

## Abstract

**Introduction:**

Adaptive immune activation in lymph nodes requires rare antigen-specific naïve T cells to locate antigen-bearing dendritic cells within a spatially structured stromal network. Reduced priming efficiency is typically attributed to weak T cell receptor signaling, yet it remains unclear whether failure arises from impaired signaling or from limited access to antigen-bearing dendritic cells during early scanning.

**Methods:**

We developed COORDINATE, a spatially explicit agent-based model of lymph node microanatomy that integrates fibroblastic reticular cell topology, chemokine-guided migration, and competition for dendritic cell access.

**Results:**

We show that stromal architecture and trafficking biases strongly influence which T cells encounter antigen, while competition for limited dendritic cell access can exclude a substantial fraction of cells from forming any productive contact. Consequently, reduced activation can arise from failed clonal recruitment rather than diminished signaling following contact.

**Discussion:**

These results support a view of early T cell priming as an access-limited process and indicate that commonly used endpoint measurements can conflate failure to access antigen with failure to activate. Together, our findings suggest that improving early antigen access, rather than strengthening signaling alone, may represent an alternative strategy to enhance adaptive immune responses.

## 1 Introduction

The lymph node (LN) is the central hub of adaptive immune activation, orchestrating antigen capture, cell trafficking, and T cell priming under finely tuned spatial and temporal constraints (1, 2). Within this microanatomically structured environment, productive naïve T cell priming requires the synchronous integration of multiple rate-limiting events: (1) dendritic-cell ingress and licensing with sustained presentation of peptide-major histocompatibility complex (pMHC) ligands; (2) chemokine-guided positioning and dwell time of rare cognate T cell clones within fibroblastic reticular cell (FRC) networks; (3) intact stromal and lymphatic-vascular architecture enabling encounter probabilities; (4) permissive cytokine and costimulatory milieu; and (5), precise spatiotemporal synchronization of the above processes to surpass activation thresholds and commit cells to nonlinear clonal expansion (1–11). Therefore, adaptive immune activation within the lymph node is governed not only by cognate pMHC availability, but also by lymph node-regulated trafficking and microanatomical organization (1–4). These features together determine whether T cell receptor (TCR)–pMHC encounters attain sufficient duration and signaling competence to drive stable T cell activation (4, 7, 12). Together, these constraints limit productive signaling to narrow time windows (4, 9, 12). Even small perturbations in antigen encounter dynamics or signaling thresholds can prevent T cells from crossing the activation threshold required for stable commitment (10, 11). While reduced priming efficiency is often attributed to insufficient TCR signaling (7), it remains unresolved whether failed activation predominantly reflects impaired signaling during T cell-dendritic cell interactions (4, 12) or limited spatiotemporal access to antigen-bearing dendritic cells.

Two-photon intravital microscopy has yielded quantitative insight into lymph node cellular dynamics, including T cell motility patterns, transient and stable T cell-dendritic cell contacts, and lymphocyte organization relative to stromal scaffolds (3, 4, 12, 13). These studies reveal marked compartment-specific heterogeneity in dendritic cell localization and intranodal trafficking, including regulated migration, positioning, and residence during T-cell priming (1, 6, 14). Despite preserving physiological context, *in-vivo* imaging is inherently confined to limited fields of view and finite observation intervals, restricting reconstruction of full migratory trajectories, complete contact histories, and integrated signaling dynamics across the priming window (15, 16). In parallel, reductionist and microphysiological platforms enable precise control over individual parameters, but they typically simplify or only partially recapitulate the intact stromal topology and compartmentalized geometry that organize immune cell encounters *in vivo* (17, 18). Thus, existing experimental approaches elucidate the T cell priming steps, yet how these steps are integrated across the lymph node to regulate priming efficiency remains unresolved.

Computational modeling provides a complementary means to examine lymph node immune priming kinetics across conditions and timescales that are experimentally inaccessible (17, 19). Such models allow explicit control and systematic perturbation of spatial organization and cellular heterogeneity, while preserving single-cell-level tracking of interaction histories (19–21). Deterministic and population-averaged models capture aggregate trends in antigen availability and mean activation dynamics but fail to resolve the spatial heterogeneity, cellular-level variability, and rare cognate-encounter structure that shape naïve T cell fate commitment (17, 22, 23). Network and compartment-based models incorporate coarse lymph node routing yet lack explicit representations of cell-level motility, contact histories, and local spatial competition (19, 24–26).

Agent-based models (ABMs) address these limitations by explicitly resolving individual cells, stochastic migration, and contact-dependent signaling within spatially structured domains (20, 21). Early ABMs of lymph node priming reproduced key features of immune activation, including contact-duration distributions, activation thresholds, and clonal expansion dynamics, but represent cell motility using random-walk or lattice-based rules that fail to encode stromal topology and spatial routing constraints (20, 21, 26). Preliminary FRC-constrained ABMs introduce spatial scaffolds, implemented as three-dimensional cellular automata or edge-vertex graphs, that restrict lymphocyte migration to filamentous paths, couple movement to chemokine-biased persistence, and enable controlled perturbation of network structure to quantify effects on trajectories and encounter rates (27, 28). However, these frameworks reduce the FRC network to a static geometric substrate rather than a topologically organized transport system. As a result, they fail to encode small-world connectivity, hierarchical routing, or anatomically defined entry-exit structure, features shown to govern lymph node organization and regulate immune-cell access (19, 29, 30).

**Figure 1:**
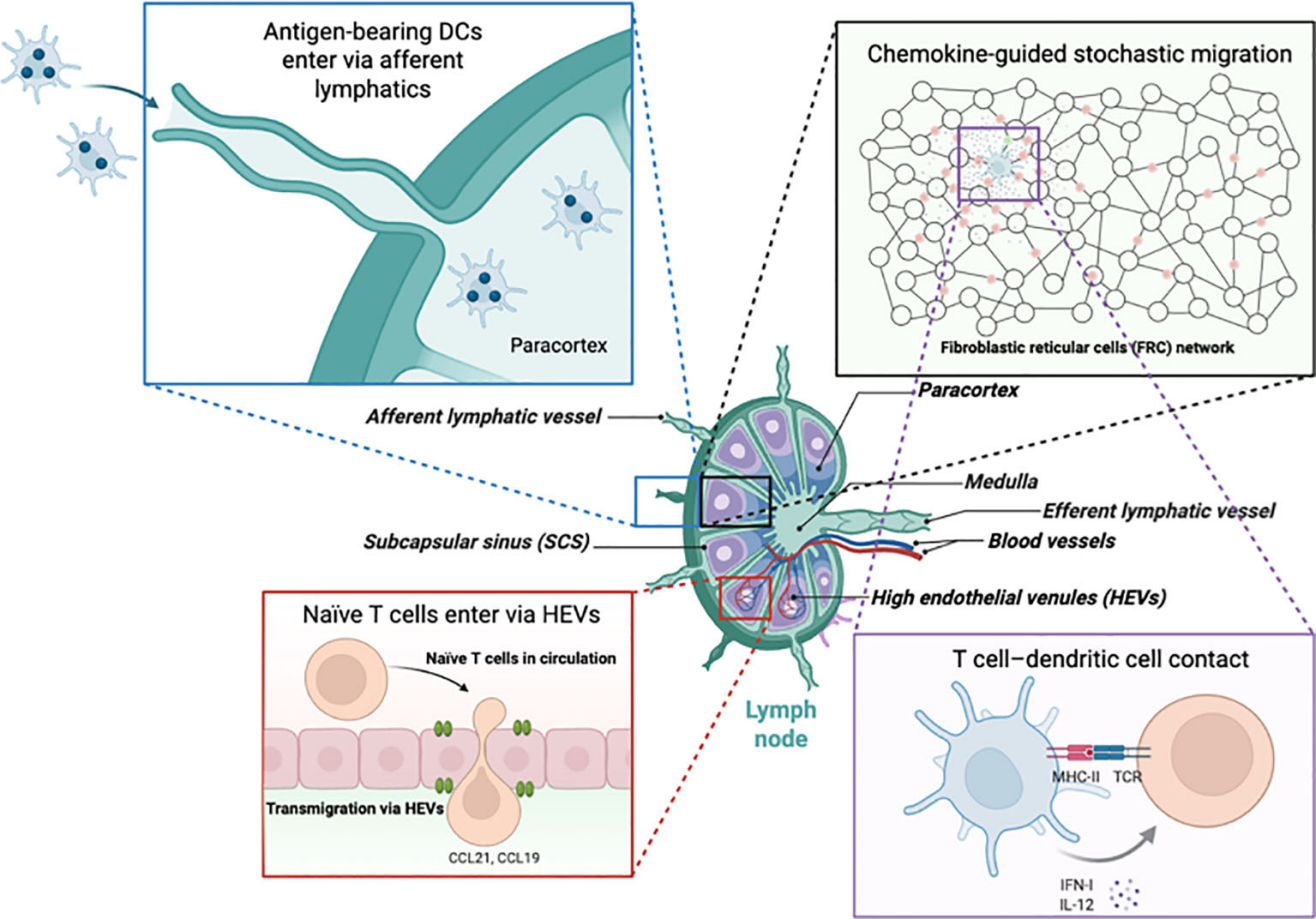
Early T cell priming is limited by spatial access in the lymph node. Dendritic cells and naïve T cells enter through distinct routes and converge within the paracortex, where chemokine-guided migration governs encounter dynamics. Productive priming depends on physical access to antigen-bearing dendritic cells. Created with BioRender.com.

Lymph node priming can therefore be viewed as a reliability challenge, in which a spatially distributed tissue must integrate stochastic cell migration, rare encounters, and time-limited signals to robustly commit a small number of cognate T cells despite substantial biological noise (4, 10–12). In the present work, we develop COORDINATE, a spatially explicit agent-based model of LN microanatomy that integrates fibroblastic reticular cell topology, chemokine-guided migration, and competitive interactions for dendritic cell access. Using controlled in silico perturbations, we disentangle how spatial topology and competition differentially shape early T cell priming dynamics. Specifically, we quantify how stromal organization biases the timing of productive encounters, while competitive constraints regulate which T cells successfully access antigen. This framework enables mechanistic interpretation of LN priming as a process governed by temporally gated encounter opportunities and competitive allocation (**Figure 1**).

## 2 Materials and methods

**Figure 2:**
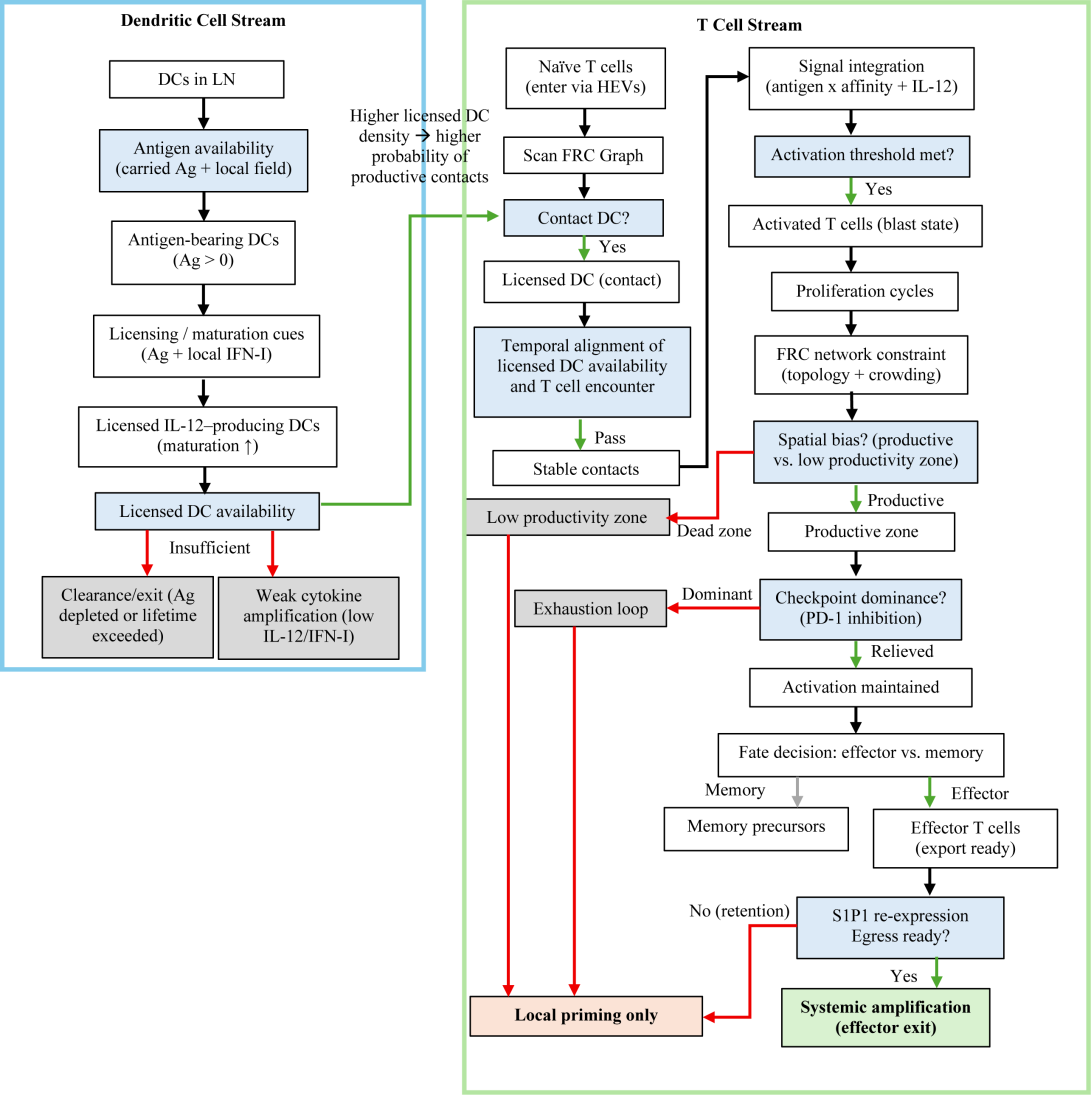
Agent-based model framework overview.

Agent-based models (ABMs) can be used to represent complex biological systems by explicitly defining interacting entities (“agents”), their environment, and the rules governing their behaviors and interactions. Our model consists of three core components: (i) a fixed three-dimensional (3D) spatial scaffold representing the lymph node FRC; (ii) discrete immune-cell agents, including dendritic cells spanning immature to licensed states, as well as naïve, activated, and differentiating T cells; and (iii) spatially distributed molecular fields representing antigen availability and cytokine-mediated signaling, including antigen, type I interferon (IFN-I), interleukin-12 (IL-12), and, chemokine CXCL10. Interactions among these components give rise to emergent system-level behaviors, including clonal expansion, exhaustion, and effector deployment (**Figure 2**).

The model was implemented as a fully custom hybrid agent-based framework in Python, using NumPy for numerical computation, NetworkX for graph-based stromal topology, and SciPy for accelerated spatial queries. This custom implementation enabled explicit control over stromal geometry and tightly coupled agent-field dynamics that are not natively supported in many general-purpose ABM platforms and typically require substantial customization.

### 2.1 Simulation framework

Time was discretized into uniform steps of duration Δt = 1 min, consistent with experimentally observed timescales of lymphocyte migration and T cell-dendritic cell contact dynamics within lymph nodes (4). Simulations were initialized at t = 0 and advanced for a total duration T (12h), spanning early scanning and stable T cell- dendritic cell interactions. The stromal network remained static throughout each simulation run, while agent populations and molecular fields evolved dynamically. At each timestep, system outcomes, such as population of immune agents, spatially distributed molecular fields, and a set of accumulated summary metrics were computed.

Each simulation step proceeded through an ordered sequence: (i) stochastic ingress of naïve T cells through HEV-associated regions; (ii) agent perception of local molecular cues, neighborhood density, and potential interaction partners within fixed spatial sensing radii; (iii) internal agent-level decision updates governing state transitions and interaction commitments; (iv) physical execution of actions, including migration along the stromal network, formation or release of cell-cell contacts, cytokine secretion, and downstream fate transitions; (v) numerical update of molecular fields via diffusion and decay; (vi) removal of dead or egressed agents; and (vii) recording of system-level metrics. All stochastic processes used fixed-seed pseudo-random number generators, with separate independent random number streams assigned to topology generation, agent behavior, molecular field updates, and event timing to ensure reproducibility.

### 2.2 Stromal representation of the lymph node

The lymph node stromal scaffold was represented as a weighted graph embedded in a 388 × 388 × 388 µm³ 3D domain (**Supplementary Figure 1A**). The paracortical FRC network was matched to published 3D T-zone FRC reconstructions (29), with surrounding compartments added using fixed relative proportions (**Supplementary Table 2**). Nodes corresponded to FRC junctions with spatial coordinates within the simulation domain, and edges represented conduit segments along which immune cells migrated. Edge lengths were defined by Euclidean distance between connected nodes. Nodes were annotated with anatomical compartment labels corresponding to the subcapsular sinus (SCS), paracortex, medulla, high endothelial venules (HEVs), and medullary exit portals. Paracortical nodes were seeded using a Poisson-disk-like sampling scheme enforcing a minimum inter-node spacing consistent with experimentally observed conduit densities (29). Local connectivity was established using k-nearest-neighbor (kNN) wiring subject to strict edge-length constraints, preventing unrealistically long conduits while maintaining global connectivity. Sheet-like node layers were generated to represent the SCS and medullary regions and were vertically bridged to the paracortical network. HEVs were represented as vertical node columns spanning the paracortex, providing spatially localized entry points for naïve T cells. Medullary exit portals were placed near domain boundaries and connected to nearby stromal nodes, defining spatially explicit egress routes.

To capture heterogeneity in the stromal microstructure, each conduit edge was annotated with stochastic microstructural properties, including effective radius, porosity, stiffness, and chemokine flux bias (**Supplementary Figures 1B–D**). These edge-level properties defined local conduit permissiveness for cell migration along the stromal scaffold. Edges with larger effective radius or higher porosity permitted faster T-cell and dendritic-cell traversal, whereas higher effective stiffness imposed a local motility penalty and reduced traversal speed. A subset of high-degree nodes was designated as dendritic cell docking hubs, reflecting preferential dendritic cell localization at highly connected stromal junctions.

### 2.3 Definition of molecular fields and boundary conditions

Cytokine and chemokine signaling was represented by continuous molecular fields defined over the three-dimensional lymph-node tissue volume. Fields included IL-12, IFN-I, and CXCL10, with antigen availability represented through dendritic-cell antigen load and local source dynamics. Antigenic stimulation was represented as a single effective pMHC signal, with T cells differing in affinity and dendritic-cell antigen load serving as a proxy for effective pMHC presentation strength. Each field was discretized on a uniform Cartesian grid with spatial resolution of 20 µm. Molecular fields evolved according to reaction-diffusion dynamics with first-order decay and additive source terms supplied by agents: $\frac{\partial F\left(x,t\right)}{\partial t}=D\nabla^{2}F\left(x,t\right)-kF\left(x,t\right)+\underset{i}{\sum }S_{i}\left(x,t\right),$ where *F* denotes the local concentration of a molecular species, *D* denotes the effective diffusion coefficient, *k* denotes the decay rate, and *S_i_* represents the contribution of individual secreting immune-cell agents, summed over all such agents at time t. At each time step, agent-derived production and uptake updated the local molecular fields, and field values contributed to migration bias, dendritic-cell maturation, T-cell signaling, and local activation-state transitions. To isolate the effects of stromal topology and cell-routing behavior, diffusion coefficients and decay rates were specified as effective tissue-scale transport parameters across molecular fields, with *D* = 60.0 *μ*m^2^s^−1^ (31) and *k* = 0.008 min^−1^ (32) (**Supplementary Table 2**). Numerical stability of the explicit 3D finite-difference scheme was ensured independently through the chosen spatial and temporal discretization.

### 2.4 Definition of immune-cell agents

#### 2.4.1 Dendritic cells

Dendritic cells were initialized at random positions on the stromal network with heterogeneous antigen loads and lifetimes. Dendritic cells migrated along the FRC network with baseline speeds sampled uniformly from 2–6 µm/min (33), with direction biased by local antigen and chemokine gradients. Upon reaching a node, outgoing edges were selected probabilistically using a softmax weighting of the cosine alignment between each candidate edge direction and the local molecular-gradient vector. Dendritic cell maturation was modeled as a continuous variable evolving according to locally integrated antigen burden and IFN-I exposure. Antigen was consumed multiplicatively over time, with consumption rates increasing as a function of cumulative contact burden and recent interaction history. Each dendritic cell maintained a finite contact capacity, limiting the number of simultaneous T cell interactions and introducing competition among responding T cells. Dendritic cells secreted IL-12 and type I interferon (IFN-I) (8, 34) into the local molecular fields, with secretion rates dependent on their maturation state and current contact load, and varying dynamically over the course of maturation. Recent contact history was represented as a transient contact-memory signal, capped at 60 minutes, that modulate cytokine secretion dynamics. Dendritic cells were removed from the simulation upon antigen exhaustion or upon reaching a predefined lifespan.

#### 2.4.2 T cells

Naïve T cells entered the system stochastically through HEV-associated regions according to a Poisson process. Each entering T cell was assigned a unique clonal identity and an antigen affinity drawn from a heavy-tailed distribution, reflecting the rarity structure of antigen-specific precursor populations. Clone-level lineage statistics, including division counts and fate outcomes, were tracked throughout the simulation. T cells migrated along the FRC network, with baseline speeds sampled uniformly from 10–12 µm/min (33). Molecular guidance cues biased directional route choice along the network: at each node, outgoing edges were selected probabilistically using a softmax weighting of their cosine alignment with the local molecular-gradient vector. Chemokine cues therefore affected direction of travel without directly rescaling baseline speed, while local crowding and conduit-level microstructural properties modulated realized displacement. Each T cell occupied a discrete phenotypic state (naïve, activated, blast, effector, memory, or exhausted), with state transitions governed by internally accumulated activation signals, checkpoint regulation, and state-specific thresholds. Progression through activation states was history-dependent. Naïve T cells transitioned to an activated state upon exceeding an activation threshold, while sustained signaling drove progression to a blast state and entry into the cell cycle. Clonal expansion occurred through successive divisions, with daughter-cell fates probabilistically biased toward effector or memory lineages according to the local cytokine environment. Terminal exhaustion could arise stochastically from the activated or blast states under conditions of excessive checkpoint signaling.

#### 2.4.3 T cell-dendritic cell interactions

T cells formed transient physical contacts with nearby antigen-bearing dendritic cells within a defined interaction radius. Contact formation was competitive and probabilistic, favoring dendritic cells with higher antigen loads and maturation states, and was constrained by a finite dendritic cell contact capacity that enforced competition for access to antigen-presenting cells. During contact, T cells accumulated activation signals as a continuous function of dendritic cell antigen load, dendritic cell maturation state, intrinsic T cell affinity, and local IL-12 concentration, with penalties imposed by local crowding estimated from the number of neighboring T cell within a local sensing radius. Contact duration was explicitly tracked and contributed to cumulative signal integration. Local cytokine exposure was sampled continuously during dendritic cell contact. IL-12 and IFN-I modulated downstream differentiation bias by influencing fate probabilities during subsequent divisions but did not directly trigger state transitions. Dendritic cell-T cell interactions were transient and released stochastically, with prolonged or repeated contacts increasing cumulative signaling load and local checkpoint pressure, thereby indirectly shaping exhaustion risk through mechanisms defined at the T cell state level. T cells remained motile during dendritic-cell engagement, with accumulated signal stabilizing contact and local crowding promoting detachment, while prolonged or repeated contacts increased cumulative signaling, checkpoint pressure, and exhaustion risk.

#### 2.4.4 Egress and effector deployment

Effector and memory T cells became eligible for egress only after satisfying biochemical, temporal, and spatial criteria, including recovery of sphingosine-1-phosphate receptor-1 (S1P1) expression, absence of recent contact, low IL-12 exposure, and proximity to medullary exit portals. Egress occurred probabilistically, after which cells were removed from the simulation and recorded as deployed effectors.

### 2.5 Case study simulations

Early T cell priming in the lymph node depends on whether naïve T cells gain timely access to antigen-bearing dendritic cells, yet the spatial, temporal, and competitive constraints that regulate this access remain difficult to disentangle experimentally. To isolate how these modeled constraints shape early priming dynamics, we performed a series of controlled in silico case studies reflecting canonical experimental perturbations. For this analysis, modeled constraints were defined as mechanisms that restrict when and where naïve T cells access antigen-bearing dendritic cells within the early priming window: stromal network topology, chemokine-guided routing, anatomical entry through HEV-associated regions, dendritic-cell availability, finite dendritic-cell contact capacity, local crowding, antigen-dependent dendritic-cell maturation, and the finite duration of early scanning. Across all case studies, cellular rules, signaling models, molecular field dynamics, interaction thresholds, and stochastic update rules were held fixed unless explicitly stated, enabling mechanism isolation. All simulations were run for 12 h with 20 independent replicates per condition, with system state recorded every 20 min unless otherwise specified. Convergence of condition-level estimates was assessed via replicate subsampling, confirming that key outcome metrics and condition-level rankings stabilized within this replicate depth (**Supplementary Figure 2**). All metrics were recorded at every timestep and exported as structured CSV files for downstream analysis.

#### 2.5.1 Case study 1: stromal network topology and migration routing

Case Study 1 examined how stromal network topology and migration routing shape encounter opportunity and early naïve T cell priming within the lymph node paracortex. We considered four environments spanning increasing biological realism: (i) a spatially regular lattice, (ii) a degree-preserving rewired network lacking spatial embedding, (iii) an anatomically structured FRC network with unbiased graph-based migration, and (iv) the same structured network with chemokine-biased, directionally guided migration (**Supplementary Figure 3**). Cases (iii) and (iv) represent biologically plausible lymph node organization, with Case (iv) most closely reflecting *in vivo* behavior due to chemokine-guided motility. The unstructured control was generated by applying degree-preserving double-edge swaps to the structured FRC network, retaining node positions, node/edge counts, degree sequence, mean degree, and nearest-neighbor spacing while randomizing connectivity. In contrast, Cases (i) and (ii) serve as geometric and topological controls, respectively, enabling isolation of spatial structure and connectivity effects. Antigenic stimulation was represented as a single effective pMHC signal, with T cells differing in affinity and dendritic-cell antigen load serving as a proxy for effective pMHC presentation strength.

#### 2.5.2 Case study 2: spatial competition for APC access

Case Study 2 probed the capacity limits of antigen-presenting cell (APC)-mediated priming. Stromal topology, biochemical field dynamics, and intrinsic activation and exhaustion rules, were held constant, while competitive pressure was introduced by systematically varying cellular load and spatial congestion. Dendritic cell availability was modulated by scaling the number of seeded dendritic cells relative to baseline (Δ_DC_ ∈ {−50%, −25%, 0%, +25%, +50%}), preserving the dendritic cell seeding protocol. T cell demand was independently modulated by scaling the naïve T cell influx rate (Δ_T_ ∈ {−50%, 0%, +50%, +100%}). Local crowding sensitivity was controlled by scaling the spatial neighborhood used to estimate local T cell density (Δ_crowd_ ∈ {−50%, 0%, +50%}), with the resulting crowding signal applied as a penalty on effective signaling accumulation and contact stability. Outcomes included exclusion (failure to achieve at least one productive dendritic cell contact), activation and exhaustion fractions, dendritic cell utilization and saturation, inequality in integrated signaling across T cells, and the spatial localization of contacts relative to the antigen hotspot. To attribute these outcomes to specific mechanisms, we fit standardized linear models to independent replicate-level outputs. Predictors were grouped into three classes: competitive design variables, capacity/access variables, and per-contact timing variables. Model explanatory power was summarized using full-model R², and individual predictor contributions were quantified using drop-column partial R², defined as the decrease in model R² after removing each predictor from the full model.

## 3 Results

COORDINATE (Computational Organization of Rare Dynamics in Network-embedded Adaptive Tissue Environments) was implemented in Python as a custom ABM for spatiotemporally resolved immune agents interacting through diffusive molecular fields. Simulations advanced in discrete 1-min time steps, with agent behaviors, cell-cell interactions, and field updates evaluated synchronously. Unless otherwise stated, simulations were run for 12 h of simulated time, sufficient to capture early T cell priming and early transition toward blast-associated behavior, while minimizing confounding effects from later-stage antigen loss or APC depletion (4, 12).

The generated paracortical FRC subgraph formed a single connected component with high local clustering and short characteristic path lengths (mean degree 8.4 ± 1.4; clustering coefficient ≈ 0.43). To assess global network organization, we computed the small-world index ω, defined relative to equivalent lattice and random networks (ω ≈ 0 indicates small-world structure; ω< 0 indicates lattice-like bias). The network yielded ω = −0.26, consistent with experimentally reconstructed T-zone FRC networks (29). Mean nearest-neighbor node spacing was 16.84 µm, closely matching the 17 µm mean FRC spacing reported *in vivo* (3).

### 3.1 Spatial topology and routing bias shape early T cell-dendritic cell priming.

**Figure 3:**
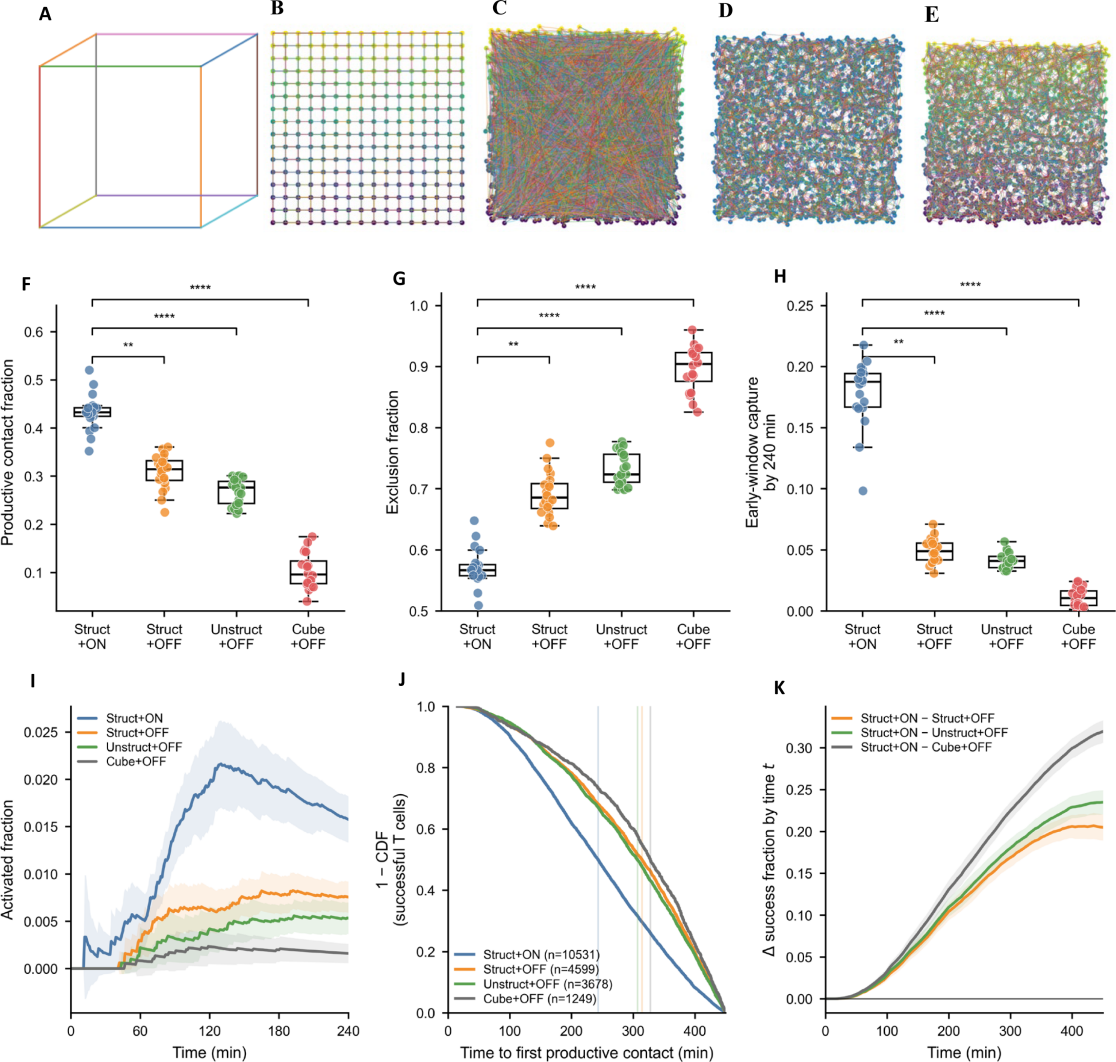
Case study 1: stromal topology and migration routing shape early T cell priming dynamics. **(A)** Cubic simulation domain used for all environments. **(B–E)** Case Study 1 migration environments. [**(B)**; Case i, Cube+OFF] Spatially regular cubic-lattice control approximating homogeneous isotropic migration in a simplified spatial domain. [**(C)**; Case ii, Unstruct+OFF] Degree-preserving rewired control that preserves the original degree distribution while disrupting anatomical spatial embedding; nonlocal edge crossings in the 2D projection illustrate the loss of local spatial organization relative to the structured FRC network. [**(D)**; Case iii, Struct+OFF] Anatomically structured, spatially embedded FRC network with unbiased graph-based migration. [**(E)**; Case iv, Struct+ON] Anatomically structured FRC network with chemokine-biased routing enabled, coupling FRC-constrained migration to local chemokine cues. **(F–H)** Replicate-level distributions for productive contact fraction, exclusion fraction, and early-window capture fraction by 240 min, respectively. Significance brackets indicate pairwise comparisons relative to Struct+ON; **p< 0.01, ***p< 0.001, ****p< 0.0001. **(I)** Activated T cell fraction over time; shaded regions denote inter-replicate variability. **(J)** Conditional timing of first productive contact among successfully contacted T cells. **(K)** Time-resolved effect size relative to comparator environments.

Our first goal was to examine how explicit stromal topology shapes T cell-dendritic cell encounter dynamics during the early priming window. Simulations in a structured lymph node environment incorporating an explicit FRC network and chemokine-guided migration were compared with unstructured environments lacking spatial embedding and directional cues, while holding all cellular rules and parameters constant (**Figures 3A–E**).

Structured stromal networks with chemokine guidance produced the highest productive contact fraction, whereas disabling chemokine guidance reduced productive encounters and both unstructured and geometric cube controls converged to lower contact outcomes (**Figure 3F**). This increase in productive contact was mirrored by a corresponding reduction in exclusion from productive encounters (**Figure 3G**). Structured networks lacking chemokine guidance still outperformed unstructured controls, suggesting that spatial embedding of the stromal scaffold can confer a priming advantage even in the absence of directed chemokine routing. However, absolute endpoint differences were modest, indicating that stromal organization does not act as an unbounded amplifier of priming success.

Time-resolved analyses revealed that these differences arose early, rather than accumulating gradually over time. Structured environments departed from baseline activation sooner and transiently accumulated activated T cells more rapidly than chemokine-off and unstructured controls, with partial convergence at later times (**Figure 3I**). Consistent with this, structured networks exhibited a higher probability of capturing a first productive T cell-dendritic cell encounter within the early temporal window by 240 min (**Figure 3H**), consistent with preferential early access to productive encounters.

To distinguish endpoint differences in encounter access from differences in encounter timing, we examined survival distributions of time to first productive contact conditioned on successful contact. Chemokine-guided structured networks shifted successful T cells toward earlier first productive contacts, as indicated by the faster decline of the Struct+ON survival curve relative to the chemokine-off, unstructured, and cube controls (**Figure 3J**). Thus, stromal topology and chemokine routing did not only increase the number of productive interactions, but they also advanced the timing at which successful T cells entered productive contact. Importantly, because post-contact activation rules were identical across conditions, these differences were best interpreted as changes in productive encounter access and timing rather than changes in intrinsic activation kinetics.

This interpretation was reinforced by time-resolved effect size analysis, which showed that Struct+ON progressively accumulated a higher success fraction than each comparator environment (**Figure 3K**). The advantage was largest relative to the cube control and remained evident relative to both Struct+OFF and Unstruct+OFF, indicating that chemokine-guided routing adds to the effect of spatial embedding. Structured chemokine-guided migration thus generated an early access advantage whose cumulative consequences remained evident across the priming window.

Together, these results support an encounter-access gating interpretation of early T cell priming. Lymph node architecture reshapes not only how many T cells enter productive dendritic cell contacts, but also when successful contacts occur. Stromal topology and chemokine-guided migration increase productive contact fractions, reduce exclusion from productive encounters, and shift successful contacts earlier in the priming window. Consequently, downstream activation differences arise from spatially organized access to productive encounter opportunities, rather than from a uniform amplification of intrinsic priming efficiency. These findings suggest that explicit representation of FRC topology is critical for capturing the temporal structure of encounter opportunities, as simplified or unstructured environments fail to reproduce this early gating behavior.

### 3.2 Competitive constraints govern access to early productive T cell-dendritic cell encounters

We next examined how access to early productive T cell-dendritic cell encounters is allocated under competitive constraints. To isolate competitive effects, stromal topology, migration rules, and antigen presentation kinetics were held fixed while independently varying T cell demand and dendritic cell supply over a ±50% range relative to baseline.

**Figure 4:**
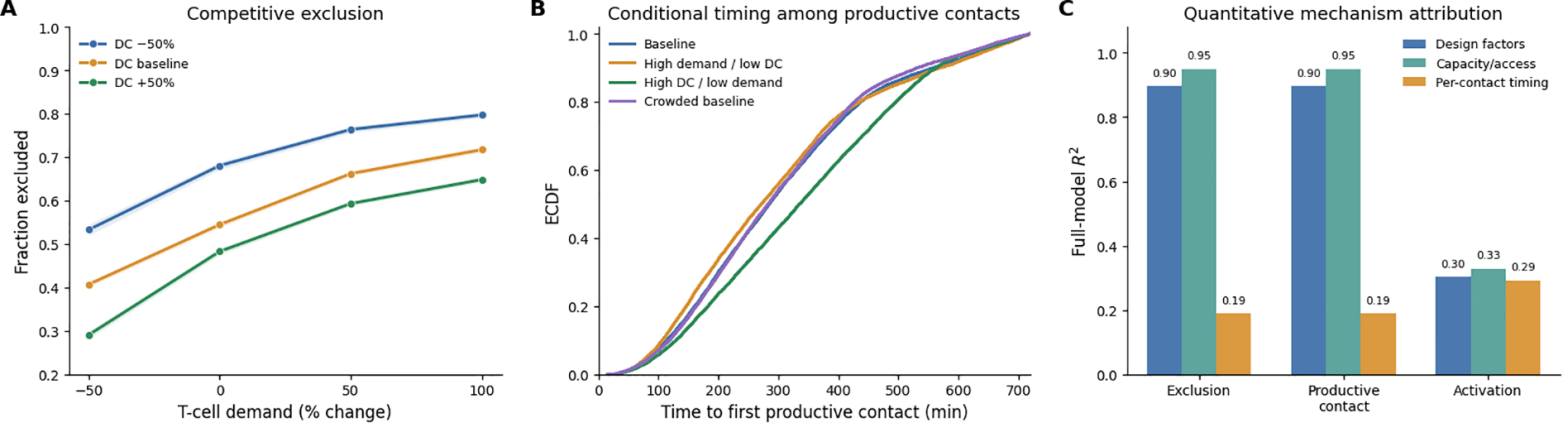
Case study 2: competitive constraints regulate productive dendritic-cell access and downstream activation. **(A)** Fraction of naïve T cells excluded from productive dendritic-cell contact as relative T-cell demand increases stratified by dendritic-cell supply. **(B)** Empirical cumulative distributions of time to first productive T cell-dendritic cell contact among T cells that formed a productive contact. **(C)** Replicate-level attribution of exclusion, productive-contact formation, and activation, showing full-model R² for design factors, capacity/access variables, and per-contact timing variables.

Across all conditions, a substantial fraction of naïve T cells failed to form productive interactions with dendritic cells within the simulation window. This indicates that productive-contact formation was strongly exclusion-limited (**Figure 4A**). Increasing T cell demand monotonically increased the fraction of excluded cells, while increasing dendritic-cell supply partially alleviated exclusion across demand levels. Notably, even under conditions of elevated dendritic-cell availability, exclusion remained prevalent, suggesting that competitive access to dendritic cells cannot be fully rescued by increasing antigen-presenting capacity alone.

These exclusion effects were nonlinear with respect to naïve T cell influx. Modest increases in T cell demand produced disproportionate increases in the fraction of excluded cells, consistent with saturation of early-access opportunities rather than gradual degradation of encounter efficiency. Conversely, reductions in T cell demand yielded diminishing returns, suggesting that competitive bottlenecks persist even under relatively sparse conditions.

To determine whether competition altered the timing of productive interactions among successful cells, we examined empirical cumulative distributions of time to first productive T cell-dendritic cell contact among T cells that formed a productive contact (**Figure 4B**). Conditional timing distributions were broadly overlapping across competitive regimes, including high-demand/low-dendritic-cell conditions and dendritic-cell-abundant/low-demand conditions. This overlap indicated that competition did not induce a distinct slow-priming regime among cells that achieved productive contact. Instead, competitive pressure primarily redistributed which T cells entered the productive-contact pool.

We then tested whether competition acted mainly by limiting access to productive contacts or by altering downstream contact timing (**Figure 4C**). Design factors, including T cell demand, dendritic-cell supply, and crowding, explained a large fraction of variation in productive-contact exclusion and productive-contact fraction (R² = 0.896). Capacity/access variables explained a similar fraction of these access outcomes (R² = 0.950), whereas per-contact timing variables explained substantially less variation (R² = 0.187). Within the design-factor model, T cell demand and dendritic-cell supply were the main upstream drivers of access, while crowding contributed less independently after demand and supply were accounted for. Activation was less strongly explained by each predictor class alone (R² = 0.304, 0.329, and 0.292, respectively), with the combined access-plus-timing model increasing explanatory power (R² = 0.500).

Together, these results suggest that competitive constraints act upstream of priming kinetics by regulating access to a limited early window of productive dendritic-cell encounters. Variations in T cell demand and dendritic-cell supply reshape population-level activation outcomes primarily by determining which cells are excluded from productive contact, rather than by uniformly altering the timing of priming among successful cells. In this regime, early stochastic access under competition strongly constrains downstream activation statistics, making endpoint activation fractions insufficient to capture the underlying allocation dynamics.

## 4 Discussion

T cell priming in LNs arises from stochastic cell-cell encounters that occur within structured tissue environments and are shaped by competition for limited access to antigen-presenting cells (4, 35, 36). These single-cell processes generate population-level outcomes that are not fully captured by endpoint measurements alone, because diverse encounter histories and early differences in access to antigen-bearing dendritic cells can be obscured once downstream activation markers are assessed (4, 37, 38). Agent-based modeling is uniquely suited to this regime because it explicitly resolves individual cells, spatial organization, and interaction timing without assumptions of homogeneity or mean-field behavior (19, 21, 39). Here, we developed COORDINATE, a lymph node agent-based model parameterized from experimental literature and evaluated without *post hoc* fitting, enabling mechanism-driven analysis. Rather than aiming to reproduce specific *in vivo* experiments, we used this framework to perform targeted case studies that disentangle regulatory mechanisms that are intrinsically conflated in experimental systems. In this role, the model functions as a hypothesis-filtering tool that helps distinguish access-based explanations from purely efficiency-based explanations within the modeled conditions, narrows the space of viable mechanisms, and generates experimentally testable predictions.

A key question in interpreting reduced T cell activation under competitive conditions is whether diminished responses reflect differences in signaling received during dendritic cell contacts or restricted access to productive dendritic cell encounters (40–42). In our simulations, across the perturbations in T cell demand and dendritic cell supply, competition behaves in the model as an upstream access constraint rather than solely as a gradual kinetic penalty. Increasing demand excludes a growing fraction of naïve T cells from forming any productive dendritic cell contacts, while the timing of first productive contact among successfully engaged cells is comparatively less affected. Consequently, population-level reductions in activation reflect an access-driven exclusion process rather than a uniform weakening of priming among successfully engaged cells. These simulations suggest that competition may act as an early allocation process, in which outcomes are determined by which T cell clones gain timely access to limited dendritic cell opportunities, not only by how efficiently contacted cells progress once engagement occurs.

These findings position FRC architecture as an active regulator of early priming dynamics rather than a passive structural scaffold (3, 39–41). By shaping migration trajectories and encounter timing, stromal topology influences which T cells gain access to antigen-bearing dendritic cells within a limited scanning window (3, 43, 44). Accurate representation of FRC organization is therefore essential for capturing the access-limited nature of priming and for ensuring that model-derived conclusions reflect biological mechanisms rather than artifacts of simplified geometry.

More broadly, our results reveal two partially distinct modes of LN regulation acting at distinct stages of priming. Stromal architecture and chemokine-guided migration regulate when productive T cell-dendritic cell encounters occur by biasing early encounter timing, whereas resource competition regulates which cells achieve any productive encounter at all by restricting access under demand. These mechanisms generate qualitatively distinct signatures: architectural organization shifts encounter distributions earlier in time, while competition truncates the responding population through exclusion. Both strongly shape downstream immune outcomes, yet their effects are only weakly reflected in endpoint-focused measurements.

Because some T cells fail to secure productive dendritic cell encounters during the early scanning period, conventional endpoint readouts can conflate failure to access antigen-bearing dendritic cells with failure to activate after contact (4, 15, 45). Endpoint measurements based on cytokine production, proliferation, or activation markers can emphasize responding cells and obscure the silent population that never engages productively (15, 45, 46). As a result, diminished immune responses are often attributed to impaired signaling or insufficient stimulus strength, when they may instead arise from early access failures imposed by spatial or competitive constraints (40, 41, 46). By explicitly tracking both engaging and non-engaging T cells, the present framework shows how substantial differences in early encounter dynamics can be masked by convergence in endpoint measurements, underscoring the need for time-resolved assessment of early T cell-dendritic cell interactions to distinguish exclusion-limited from efficiency-limited regulatory regimes *in vivo*.

An important implication is that immune interventions may be most effective when they act during the early access and scanning window, before priming trajectories are fully established (44, 47). Increases in antigen load, adjuvant potency, or dendritic cell abundance applied at later times may fail not because signaling pathways are saturated, but because access opportunities have already narrowed for a large fraction of naïve T cells (42, 48, 49). In contrast, interventions that expand access or reshape encounter timing during early scanning windows are more likely to recruit additional clones into the response. These early temporal regimes are difficult to isolate experimentally due to the transient and stochastic nature of initial encounters, making computational interrogation particularly valuable (15, 50, 51).

Several limitations of this study should be noted. The model focuses on early priming dynamics within a defined temporal window and does not explicitly represent later stages of differentiation, memory formation, or feedback from activated T cells to dendritic cells. Other mechanisms that may influence priming were intentionally omitted or simplified to preserve interpretability, including dynamic stromal remodeling, lymph flow, dendritic-cell subset specialization, B-cell follicle interactions, antigen transfer among antigen-presenting cells, regulatory T-cell suppression, and detailed receptor-level chemokine signaling. Dendritic cells are treated as a homogeneous population with fixed antigen presentation properties, and chemokine-guided migration is represented at a simplified level sufficient to bias trajectories without capturing receptor-level dynamics or additional mechanical constraints. Despite these simplifications, the model is intentionally minimal with respect to priming kinetics, enabling a clear separation between access, competition, and downstream activation dynamics.

In summary, COORDINATE shows how early T cell priming can be governed by access to productive dendritic cell encounters before downstream activation differences become apparent. Across controlled case studies, stromal topology, chemokine-guided migration, and resource competition shaped priming through distinct access-based mechanisms: architecture altered the timing and cumulative availability of productive encounters, whereas competition restricted which cells entered productive contact under elevated demand. These results support a view of the lymph node as an active spatial regulator of immune allocation, where early encounter opportunities define the population available for downstream activation. More broadly, this study illustrates how spatially resolved agent-based models can help separate access-limited from efficiency-limited mechanisms and generate experimentally testable hypotheses about early immune decision-making.

## Data Availability

All source code and datasets generated and analyzed in this study are publicly available on GitHub at https://github.com/lvancell/COORDINATE.git.
