# Supplementary material for: Spatial topology and competitive access differentially shape early T cell priming in the lymph node: an agent-based modeling approach
Source: Front Immunol. 2026 Jun 2;17:1843390. doi: 10.3389/fimmu.2026.1843390 (PMC13269276; doi:10.3389/fimmu.2026.1843390)
Supplement: Supplementary file 1 [file Table1.docx]

***Supplementary Material***

**Spatial Topology and Competitive Access Differentially Shape Early T Cell Priming in the Lymph Node**

**Laia Vancells^1^, Leopold Green^1*^, Nan Kong^1*^**

*¹ Weldon School of Biomedical Engineering, Purdue University, West Lafayette, IN, United States*

*** Correspondence:** Leopold Green: greenln@purdue.edu; Nan Kong: nkong@purdue.edu

#### **1. SUPPLEMENTARY FIGURES**

**Supplementary Figure S1.** Validation of stromal network geometry and heterogeneity.

**Supplementary Figure S2. Replicate-depth convergence of primary summary metrics.**
**Supplementary Figure S3.** Spatial embedding of stromal network topologies (Case Study 1).
**Supplementary Figure S4.** Competitive priming phase diagrams (Case Study 2).

#### **2. SUPPLEMENTARY TABLES**

**Supplementary Table S1.** Sources and derivations for agent parameter
**Supplementary Table S2.** Sources and derivation for environment parameters.

**3. SUPPLEMENTARY REFERENCES**

1. **SUPPLEMENTAL FIGURES**

A


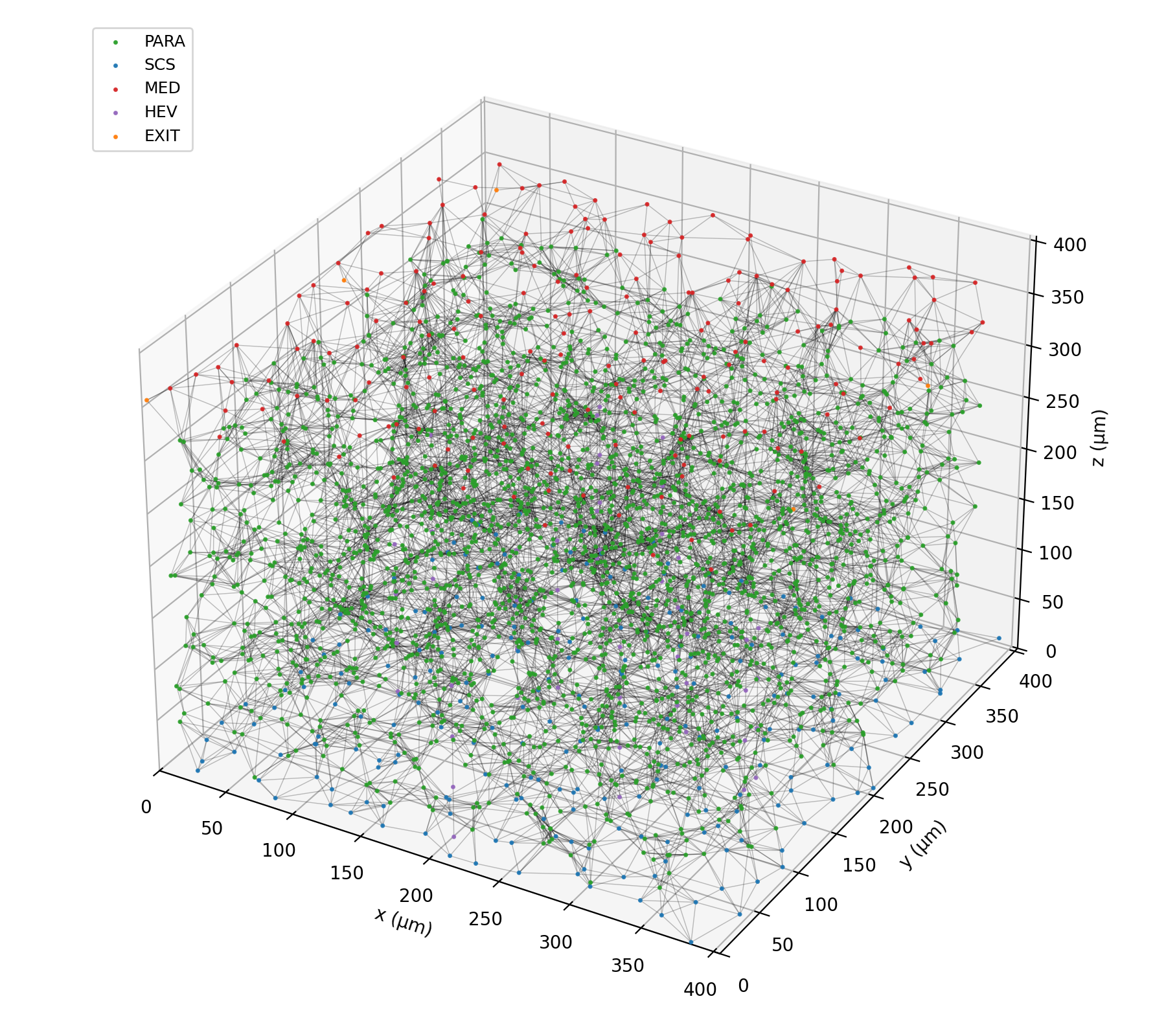


**
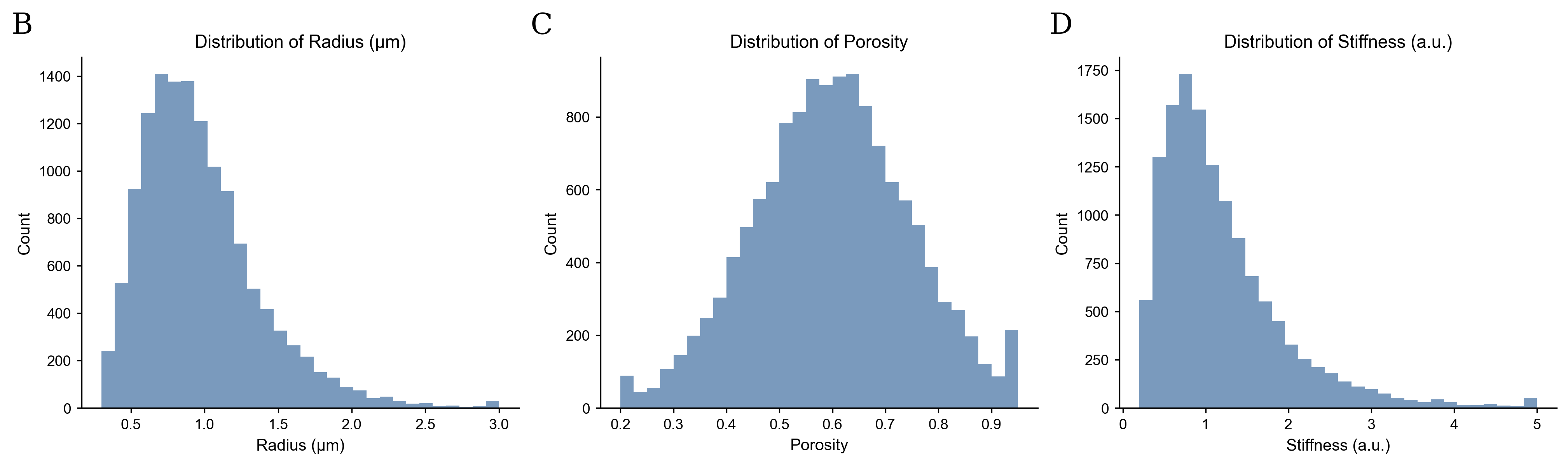
**

**Supplementary Figure S1. Validation of stromal network geometry and heterogeneity.** **(A)** Three-dimensional visualization of the FRC conduit network, colored by anatomical compartment (subcapsular sinus, paracortex, medulla, HEV entry points, and exit portals). **(B–D)** Distributions of conduit microstructural properties assigned at initialization, including effective conduit radius (**B**), local porosity (**C**), and effective stiffness (**D**). The distributions were centered near baseline traversal behavior and bounded to introduce conservative spatial heterogeneity in conduit traversal.

**
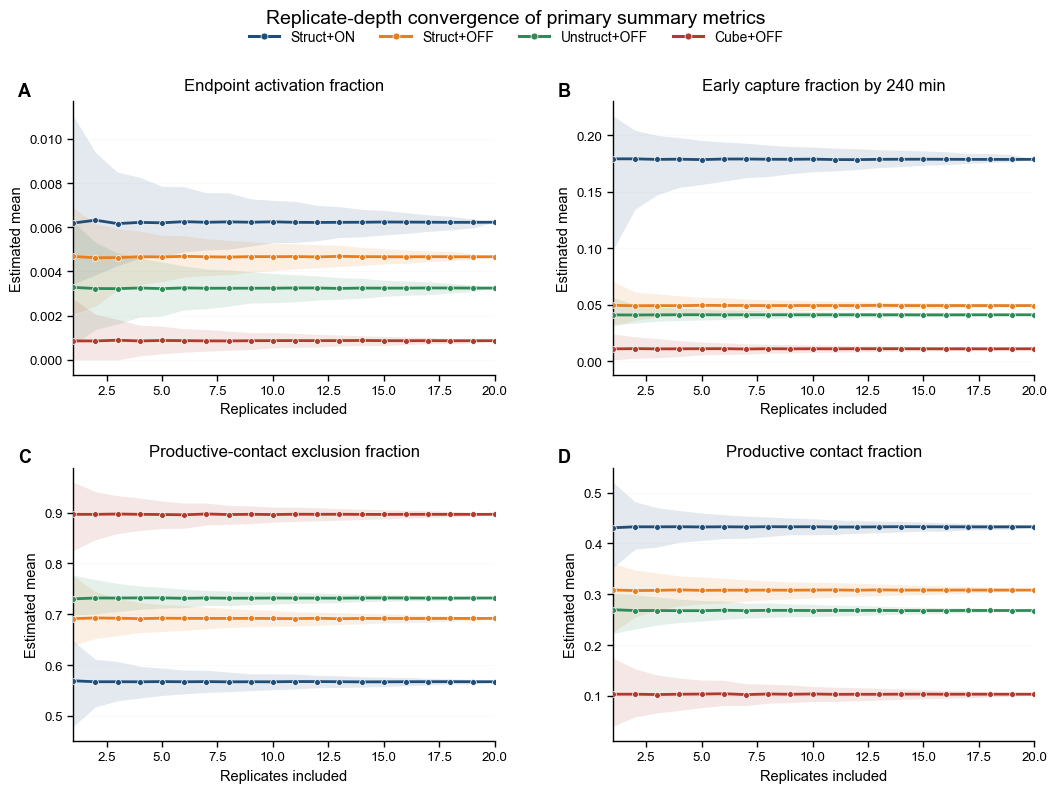
**

**
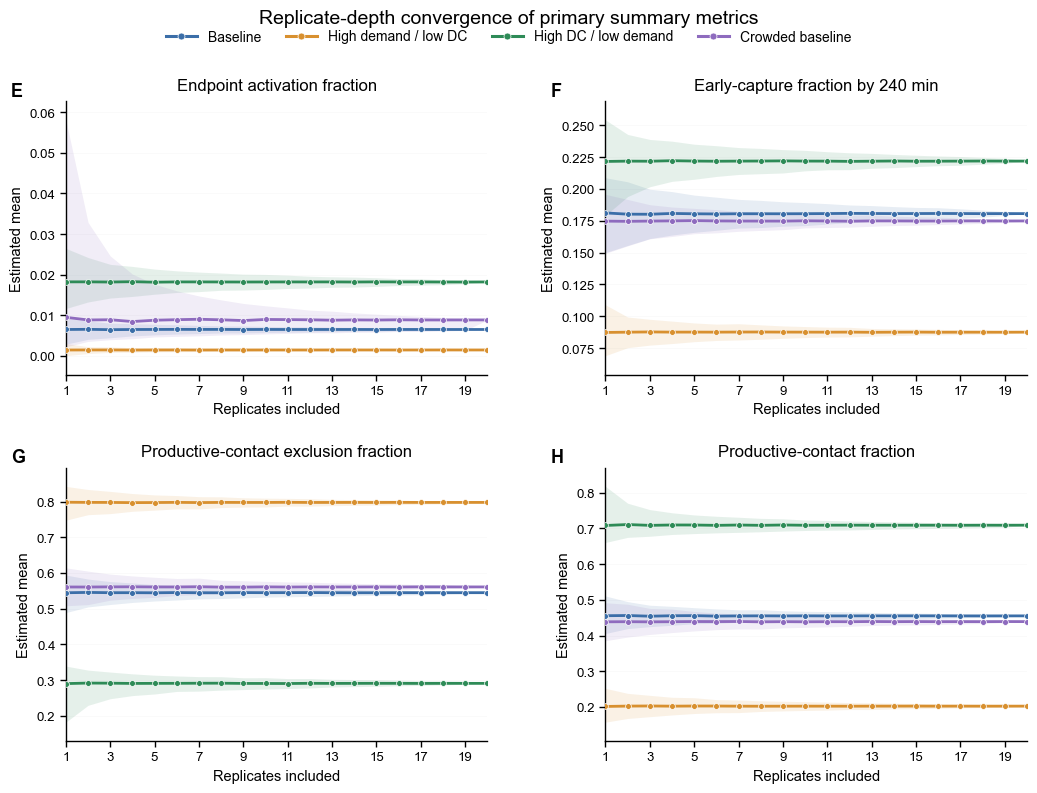
**

**Supplementary Figure S2. Replicate-depth convergence of primary summary metrics. Convergence of key outcomes was evaluated by random subsampling of independent stochastic replicates. For each condition and replicate depth, subsets were sampled without replacement and condition-level means were recomputed across repeated draws. Solid lines show the mean across subsamples, with shaded regions indicating the 95% subsampling interval. Panels A–D show Case Study 1 topology/routing conditions, and panels E–H show Case Study 2 competitive-access conditions. Rapid stabilization of the estimates indicates that the replicate depth used in the main analyses was sufficient to resolve condition-level differences.**

B

A


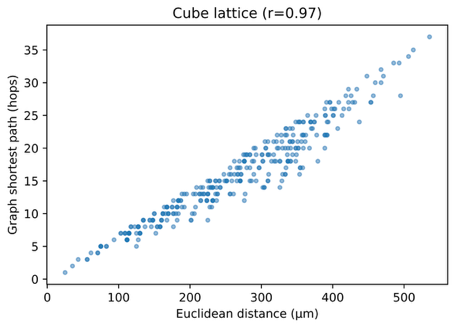

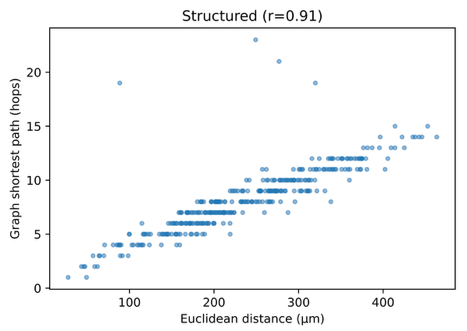


D

C

**
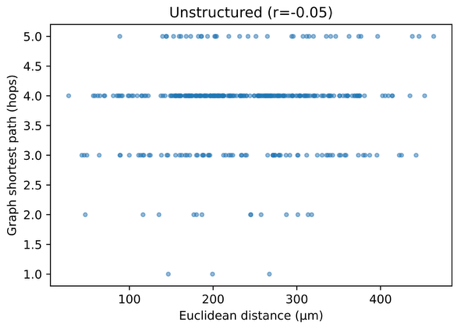
**

**Supplementary Figure S3. Spatial embedding of stromal network topologies (Case Study 1).** (**A**) Regular cube lattice showing a near-linear coupling between Euclidean distance and graph shortest-path length ($r=0.97$). (**B**) Structured FRC network preserving a strong monotonic relationship between physical separation and routing distance despite heterogeneous connectivity ($r=0.91$). (**C**) Degree-preserving unstructured (rewired) network exhibiting no systematic relationship between Euclidean distance and graph shortest-path length ($r\approx-0.05$). (**D**) Edge-length distributions distinguishing the structured FRC network from a degree-preserving unstructured topological null that removes spatial embedding to isolate routing constraints. The structured and unstructured networks both contained 2226 nodes and 9589 edges, with the same mean degree of 8.62 ± 2.00 and mean nearest-neighbor spacing of 16.84 µm. Rewiring reduced clustering from 0.410 to 0.003 and disrupted the relationship between graph distance and physical distance. For comparison, the regular cube lattice contained 4096 nodes and 11,520 edges, with mean degree 5.63 ± 0.57 and mean nearest-neighbor spacing of 25.00 µm.

**
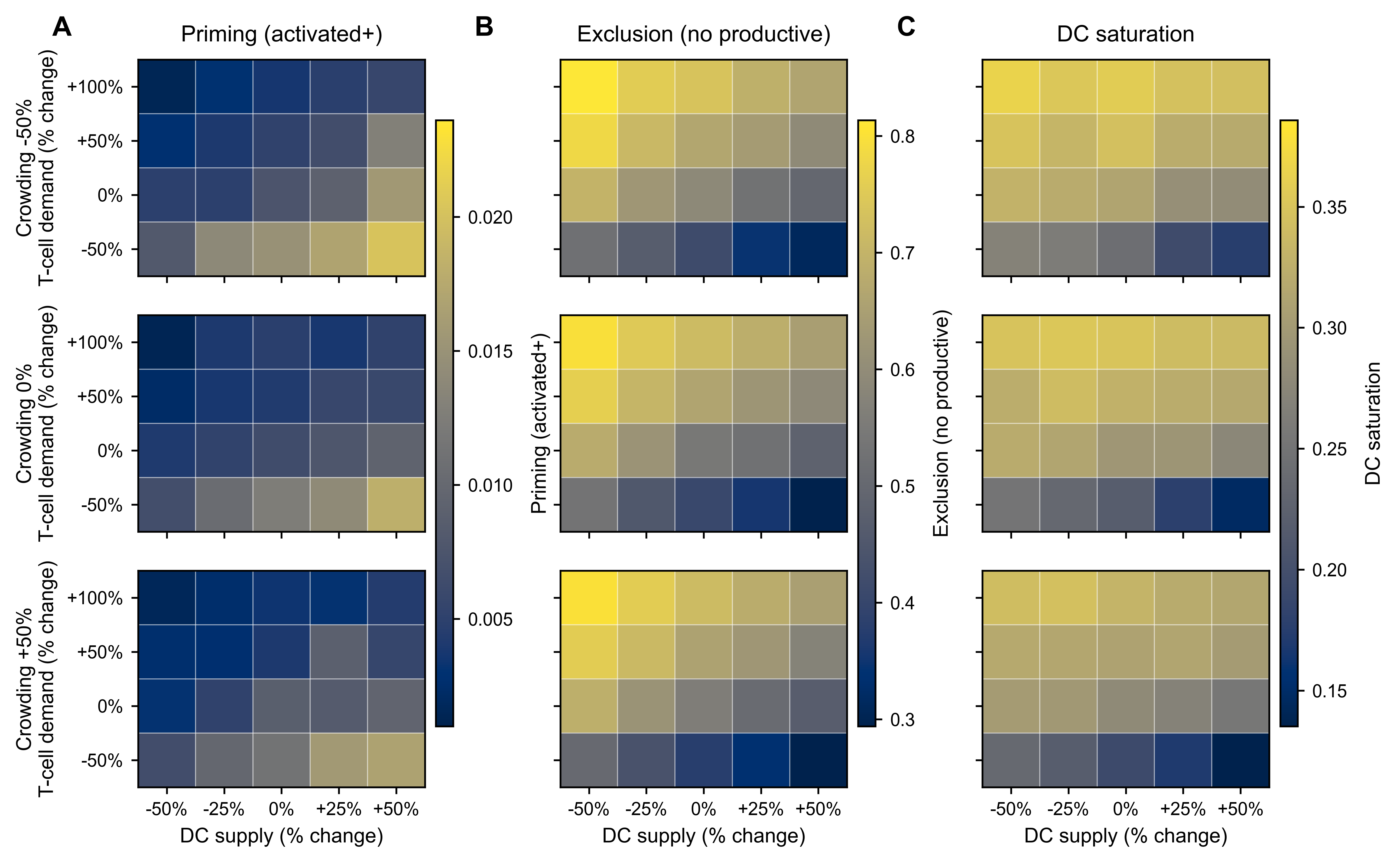
**

**Supplementary Figure S4. Competitive priming phase diagrams under varying spatial crowding (Case Study 2).** Heatmaps summarize median outcomes across replicate simulations as a function of DC supply (x-axis) and naïve T-cell demand (y-axis), stratified by crowding regime (rows). (**A**) Priming efficiency, measured as the fraction of T cells reaching activated or downstream states. (**B**) Exclusion, defined as the fraction of T cells failing to achieve any productive DC contact. (**C**) DC saturation, defined as the fraction of DCs operating at full contact capacity. Increasing T-cell demand induces DC saturation, leading to competitive exclusion and reduced priming, while reduced crowding shifts these phase boundaries without altering the underlying mechanism.

1. **SUPPLEMENTARY TABLES**

**Supplementary Table 1.** Sources and derivations for agent parameters.

| **Parameter** | **Code** | **Value*** | | **Source / Derivation** |
| --- | --- | --- | --- | --- |
| Radius for field sensing | sense_radius | 35 µm | Effective local sensing radius above the DC-T reaction scale (~25 μm), capturing stromal-neighborhood field/crowding sensing beyond direct contact (1,2). | |
| DC-T contact threshold | contact_radius | 12 µm | Cell-size-based center-to-center DC-T contact distance (sum of radii): naïve T cells ~8–10 µm and mature DCs ~10–15 µm diameter 🡪 contact distance ~9–12.5 µm; threshold set to 12 µm (3,4). | |
| Naive T-cell entries** | naive_influx_per_min | 2.0 cells/min | Volume-scaled baseline LN T-cell recruitment (2000 cells/h) to a 304-µm cube domain (5). | |
| Initial antigen-bearing DC density** | n_dcs | 308 cells/mm³ | Intermediate between the transport-limited antigen-primed DC encounter threshold (>35 cells/mm³) and DC-density regimes explored in prior virtual-LN activation models (800–8000 cells/mm³) (6,7). | |
| Background DC influx** | dc_influx_per_min | 0.03 cells/min | Turnover-balanced replenishment for the baseline DC pool: 18 seeded DCs / 600-min mean modeled lifetime = 0.03 cells/min. | |
| DC migration speed | DC.speed | 2-6 µm/min | Two-photon imaging–based DC motility (2); sampled from a uniform distribution over the range. | |
| Max T cells per DC | contact_capacity | 10 cells | Two-photon microscopy shows a mean of 1.8 ± 1.1 simultaneous CD8⁺ T cells per DC at high DC density, with >10 contacts possible (surface saturation) at low DC density (8). | |
| T-cell migration speed | TCell.speed | 10-12 µm/min | Two-photon imaging–derived T-cell motility in LN (2). | |

* Units are reported in their experimentally relevant forms; no unit normalization was applied.

** Under the unperturbed Case Study 2 reference condition, the mean modeled densities over the 12 h simulation window were 341.6 ± 118.4 dendritic cells/mm³ and 12,319.7 ± 7,031.6 T cells/mm³.

**Supplementary Table 2.** Sources and derivation for environment parameters.

| **Parameter** | **Code** | **Value** | | **Source / Derivation** |
| --- | --- | --- | --- | --- |
| Domain side length (x, y) | Lx, Ly, Lz | 388.0 µm | Estimated. Total depth (388 µm) preserves the imaging-based paracortical field-of-view (304 µm) (10). | |
| Paracortical zone fraction | paracortex_fraction | 0.78 | Estimated. Reported T-cell zone depth spans 110–625 µm (≈0.5 mm)(11); 0.78 reflects the ratio of the modeled paracortical depth (304 µm) to this extent. | |
| Subcapsular sinus thickness | scs_thickness_um | 14 µm | Measured SCS geometry in steady-state mouse LN (mean SCS height ≈ 14 µm; strand spacing ≈ 16 µm) (12,13) | |
| Medullary region thickness | medulla_thickness_um | 70 µm | Estimated. LN bottom ~740 µm and T-cell zone extent ~625 µm imply a medullary remainder of ~115 µm, proportionally scaled to the modeled paracortical depth to yield 70 µm(11). | |
| FRC topology seeding control | spacing_um | 26.6 µm | Generator input calibrated to yield 16.84 µm realized nearest-neighbor spacing, matching the reported in vivo FRC spacing of 17.26 ± 6.93 µm (range 5–37 µm) (14). | |
| Spatial heterogeneity (jitter) | jitter_um | 5.0 µm | Positional perturbation introduced to represent spatial micro-heterogeneity; magnitude chosen conservatively to match the lower bound of reported FRC inter-intersection spacing (5–37 µm; 17.26 ± 6.93 µm) (14). | |
| Small-world shortcut count | long_range_edges | 300 edges | Derived to reproduce LN small-world metrics σ ≈ 6.1 ± 0.7 and ω ≈ −0.3 (10). | |
| DC-hub fraction | dc_docking_fraction | 0.085 | Approx. 15 of 176 nodes identified as high-degree hubs in LN networks (10). | |
| HEV entry count | hev_count | 13 | Derived from HEV density ≈ 478 vessels·mm⁻³ scaled to LN volume (15). | |
| Exit portal count | exit_count | 5 | Effective absorbing exit portals; number chosen to avoid egress limitation, consistent with reported upper-bound murine LN efferent exit counts (16). | |
| Diffusion coefficient (all fields) | field_diffusion_coeff | 60.0 µm²/s | Effective LN tissue diffusion. Micro-IOI measurements report ≈67–68 µm²/s for 10 kDa dextran and ≈40–110 µm²/s across cytokine-sized solutes (17). | |
| Exponential decay constant | field_decay_rate | 0.008 min⁻¹ | Effective first-order clearance timescale for cytokine-like fields, anchored to reported terminal IL-2 kinetics ($t_{1/2}\approx85\text{ }\min\Rightarrow k\approx0.008\text{ }\mathrm{mi}n^{-1}$) and applied uniformly across fields (18). | |

1. **REFERENCES**

1. Azarov I, Peskov K, Helmlinger G, Kosinsky Y. Role of T Cell-To-Dendritic Cell Chemoattraction in T Cell Priming Initiation in the Lymph Node: An Agent-Based Modeling Study. Front Immunol. 2019;10:1289. doi:10.3389/fimmu.2019.01289 PubMed PMID: 31244840; PubMed Central PMCID: PMC6579912.

2. Bousso P, Robey EA. Dynamic Behavior of T Cells and Thymocytes in Lymphoid Organs as Revealed by Two-Photon Microscopy. Immunity. 2004 Sep;21(3):349–55. doi:10.1016/j.immuni.2004.08.005

3. Cano RLE, Lopera HDE. Introduction to T and B lymphocytes. In: Autoimmunity: From Bench to Bedside [Internet] [Internet]. El Rosario University Press; 2013 [cited 2026 Jan 19]. Available from: https://www.ncbi.nlm.nih.gov/books/NBK459471/

4. Dumortier H, Van Mierlo GJD, Egan D, Van Ewijk W, Toes REM, Offringa R, et al. Antigen Presentation by an Immature Myeloid Dendritic Cell Line Does Not Cause CTL Deletion In Vivo, but Generates CD8+ Central Memory-Like T Cells That Can Be Rescued for Full Effector Function. J Immunol. 2005 Jul 15;175(2):855–63. doi:10.4049/jimmunol.175.2.855

5. Johnson SC, Frattolin J, Edgar LT, Jafarnejad M, Moore JE. Lymph node swelling combined with temporary effector T cell retention aids T cell response in a model of adaptive immunity. J R Soc Interface. 2021 Dec;18(185):20210464. doi:10.1098/rsif.2021.0464 PubMed PMID: 34847790; PubMed Central PMCID: PMC8633806.

6. Moreau HD, Bogle G, Bousso P. A virtual lymph node model to dissect the requirements for T‐cell activation by synapses and kinapses. Immunol Cell Biol. 2016 Aug;94(7):680–8. doi:10.1038/icb.2016.36

7. Preston SP, Waters SL, Jensen OE, Heaton PR, Pritchard DI. T-cell motility in the early stages of the immune response modeled as a random walk amongst targets. Phys Rev E. 2006 Jul 17;74(1):011910. doi:10.1103/PhysRevE.74.011910

8. Bousso P, Robey E. Dynamics of CD8+ T cell priming by dendritic cells in intact lymph nodes. Nat Immunol. 2003 Jun 1;4(6):579–85. doi:10.1038/ni928

9. Kamath AT, Henri S, Battye F, Tough DF, Shortman K. Developmental kinetics and lifespan of dendritic cells in mouse lymphoid organs. Blood. 2002 Sep 1;100(5):1734–41. doi:10.1182/blood.V100.5.1734.h81702001734_1734_1741

10. Novkovic M, Onder L, Cupovic J, Abe J, Bomze D, Cremasco V, et al. Topological Small-World Organization of the Fibroblastic Reticular Cell Network Determines Lymph Node Functionality. Schroeder T, editor. PLOS Biol. 2016 Jul 14;14(7):e1002515. doi:10.1371/journal.pbio.1002515

11. Choe K, Hontani Y, Wang T, Hebert E, Ouzounov DG, Lai K, et al. Intravital three-photon microscopy allows visualization over the entire depth of mouse lymph nodes. Nat Immunol. 2022 Feb;23(2):330–40. doi:10.1038/s41590-021-01101-1 PubMed PMID: 35087231; PubMed Central PMCID: PMC9210714.

12. Arroz-Madeira S, Bekkhus T, Ulvmar MH, Petrova TV. Lessons of Vascular Specialization From Secondary Lymphoid Organ Lymphatic Endothelial Cells. Circ Res. 2023 Apr 28;132(9):1203–25. doi:10.1161/CIRCRESAHA.123.322136

13. Martens R, Permanyer M, Werth K, Yu K, Braun A, Halle O, et al. Efficient homing of T cells via afferent lymphatics requires mechanical arrest and integrin-supported chemokine guidance. Nat Commun. 2020 Feb 28;11(1):1114. doi:10.1038/s41467-020-14921-w PubMed PMID: 32111837; PubMed Central PMCID: PMC7048855.

14. Bajénoff M, Egen JG, Koo LY, Laugier JP, Brau F, Glaichenhaus N, et al. Stromal Cell Networks Regulate Lymphocyte Entry, Migration, and Territoriality in Lymph Nodes. Immunity. 2006 Dec;25(6):989–1001. doi:10.1016/j.immuni.2006.10.011

15. Kelch ID, Bogle G, Sands GB, Phillips ARJ, LeGrice IJ, Rod Dunbar P. Organ-wide 3D-imaging and topological analysis of the continuous microvascular network in a murine lymph node. Sci Rep. 2015 Nov 16;5(1):16534. doi:10.1038/srep16534

16. Kowala MC, Schoefl GI. The popliteal lymph node of the mouse: internal architecture, vascular distribution and lymphatic supply. J Anat. 1986 Oct;148:25–46. PubMed PMID: 3693091; PubMed Central PMCID: PMC1261588.

17. Ross AE, Pompano RR. Diffusion of cytokines in live lymph node tissue using microfluidic integrated optical imaging. Anal Chim Acta. 2018 Feb;1000:205–13. doi:10.1016/j.aca.2017.11.048

18. Konrad MW, Hemstreet G, Hersh EM, Mansell PW, Mertelsmann R, Kolitz JE, et al. Pharmacokinetics of recombinant interleukin 2 in humans. Cancer Res. 1990 Apr 1;50(7):2009–17. PubMed PMID: 2317789.
